# Nutritional status of school children in South-west Nigeria: Inferences from a national homegrown school feeding programme

**DOI:** 10.4314/ahs.v24i1.29

**Published:** 2024-03

**Authors:** Taiwo Akinyode Obembe, Ayoola Oluwaseun Bosede, Oluwaseun Ariyo, Folashayo I P Adeniji, Abiodun Olaoye, Ayodeji Mathew Adebayo

**Affiliations:** 1 Department of Health Policy and Management, Faculty of Public Health, University of Ibadan, Ibadan, Nigeria; 2 Department of Environmental Health Science, School of Health Technology, Federal University of Technology, Owerri, Imo State, Nigeria; 3 Department of Human Nutrition, Faculty of Public Health, University of Ibadan, Ibadan, Nigeria; 4 Department of Community Medicine, Faculty of Public Health, University of Ibadan, Ibadan, Nigeria

**Keywords:** School children, Nutritional assessment, Stunting, Underweight, Wasting, School feeding programme

## Abstract

**Background:**

The School Feeding Programme if properly executed has the capacity to improve the nutritional status of the school children.

**Objective:**

To assess the nutritional status of school children in Ondo State Nigeria given that the National Home-Grown School Feeding Programme (NHGSFP) has been operational in the state for over five years.

**Methods:**

This was a descriptive cross-sectional study.

**Results:**

A total of 234 subjects from public schools and 227 subjects from private schools were enrolled in the study. Their mean age was 8.23 ± 1.92 years. Wasting, overweight, obesity, underweight, and stunting were noted in 19.4%, 11.4%, 0.4%, 5.0%, and 20.7% of the children, respectively. The prevalence of stunting (30.3%) and wasting (23.9%) was more among subjects from the public schools. A significant association was found between Weight-for-Age Z-score, Height-for-Age Z-score, and BMI-for-Age Z-score and the children's school type (p < 0.005).

**Conclusion:**

Majority of the children showed normal growth, the rest were in both extremes of malnutrition, the subjects from private schools seem to present better nutritional status, although there is no baseline data to ratify this finding. A further study on this subject using the current finding as a baseline data is recommended.

## Introduction

School Health Programme (SHP) is designed to meet the health needs of students at the present time and lay a solid foundation for their future with the support of the home, community, and government.^1^ It is defined as, “the totality of projects and activities in a school environment, which are designed to protect and promote the health and development of the school community”.^2^ The SHP aims to promote a rapid and sustained improvement in the health of school children, and to ensure that children from preschool age to adolescence are in optimum health at all times. Despite this, 25.5%, 24.4% and 20.6% of the Nigerian school-age children are stunted, underweighted and wasted respectively.^3^ This level of malnutrition among young people is worrisome in a country like Nigeria that has a high population of adolescents (10–19 years). This is however not surprising considering that the implementation of health care services in schools has been poor. The downsides of the poor implementation have not only led to malnourishment among school children, but has also promoted other adverse consequences on school attendance, performance of the children in school, their oral well-being, and other health impacts.^4^

Nutrition is an important component of health and well-being especially for the many vulnerable populations including children and adolescents.^1^ It is not just a key determinant of physical and mental development, but also a direct reflection of the financial status of the family and the general well-being of the community, efficiency of the healthcare system, and the contribution of the general environment.^5^ Long-standing undernutrition has been reported to lead to poor intellectual growth, reduced school participation, increased school withdrawal rate, and impactful disabilities later in life; these decrease the quality of life of the affected.^6^ Similarly, overweight and obesity increase the threat of cardiovascular and lung conditions.^7^ This suggests the need to ensure adequate nutrition and sustain same for immediate and future benefits of school children in Nigeria. Pockets of studies have shown that the prevalence malnutrition among school children and in-school adolescents varied according to geographical trends.^8^,^9^ In both rich and poor nations, rise in the incidence and prevalence of overweight and obesity have been recorded among children.^10^ Dietary change from consumption of diversified meals to energy ladened bites and baked foods; coupled with lifestyle and cultural change, have been implicated in the rise in obesity among the children.^11^,^12^ Underweight (as measured by Weight-for-Age Z-score (WAZ) assessment among children less than age 10 years and Body mass index-for-Age Z-score (BAZ) assessment among the adolescents) is an indication of an ongoing malnutrition in a child. Wasting suggests an acute severe malnutrition, while stunting is an indicator of a chronic or long-standing malnutrition. The health effects on these conditions range from infectious to non-infectious diseases in the affected child. The most important effect of malnutrition that concerns this research work is the reduced cognitive and learning ability which if not promptly addressed can leave the child cognitively impaired even in adulthood.^13^ Besides, lack of adequate nutrition has also been reported as one of the important cause of school absenteeism.^14^

The National Home-Grown School Feeding Programme (NHGSFP) promoters did sufficiently justify the need for the programme, but failed to justify why the programme must be limited to government owned primary schools in Nigeria.^15^ Previous studies have also, reported varying degrees of malnutrition among the Nigeria school children, this may be the motivation for the development and implementation of the NHGSFP. A 2007 study by Oninla et al., reported over 60% prevalence of underweight among the school children in a south-western State in Nigeria.^22^ The prevalence of Stunting (an indication of chronic malnutrition) was reported as 11.1% among the school children in Plateau State Nigeria.^23^ Another study reported a 46.2% prevalence of Stunting among the school children in rural communities in Ogun State Nigeria. One may argue that it on the backdrop of these identified nutritional conditions among the school children in Nigeria that the NHGSFP was instituted.

The NHGSFP which was introduced in 2016 is a key component of SHP with the aim of delivering a government-driven, efficient food supply programme using locally grown food. The programme was designed to promote multiple benefits ranging from reduction of hunger and malnutrition, increased school enrolment, improved girl-child education, reduced drop-out rate to improved education outcomes.^15^ In spite of the laudable goal of the programme, implementation was initiated without baseline assessment of anthropometric parameters of the beneficiaries: a vital step in evaluating the impact of the programme on the overall health and well-being of school-age children. Furthermore, evidence-based impact assessment derived from a baseline assessment would be relevant for advocacy to scale up the implementation of the school feeding programme and other school health programmes.

Consequently, this study aims to create a baseline data on which the impact of the NHGSF programme and other nutritional intervention can be evaluated. Also, the anthropometric parameters of school-age children attending selected public primary schools that are participating in the Home-Grown School Feeding Programme while using selected private primary schools that are not part of the Home-Grown Feeding Programme in Ondo State Nigeria as a comparison group. This set of school children are comparable in all ways apart from the fact that the Home-Grown School Feeding Programme which is a government initiative provides school meals to children in the government owned public primary schools across the country only.

## Methods

### Study design

The study was a cross-sectional descriptive study as only a cross section of all primary schools in the study setting were observed. Even though the study was descriptive, analysis was conducted to identify the strength and direction of association between some of the study variables thereby, giving the study an outlook of analytical study.

### Study setting

The research was conducted in Akure between August 2020 and December 2020. Akure is the headquarters of Ondo State in the south-western region of Nigeria. The preponderance of commercial, industrial and educational activities in Akure is responsible for the agglomeration of people in the city. The major areas in the city where evidence of child labour is seen include, Ojo-Oba, NEPA, Road-Block/FUTA road and Arakale area.^17^

### Studied population

This research report is extracted from a larger study on the appraisal of the SHP in Ondo State, thus the children for this study were recruited from selected 42 primary schools in the State. All obviously healthy children that were found in the schools at the time of data collection were deemed eligible for the study. A minimum sample size of 426 school children was arrived at using Leslie Kish formula for determining single proportion for descriptive study, considering that prevalence “P” of 50.0% representing highest proportion of children with normal weight was utilized as a baseline for comparison at a critical ratio of 1.96 and level of precision of 5.0% Thus, an average of 11 children were randomly selected from each of the schools. Out of the total 461 selected subjects, 234 were obtained from the public schools (owned and operated by the state government) where the NHGSFP is active; the remaining 227 were obtained from private schools (owned and operated by private educational entrepreneurs) where the NHGSFP is non-existent.

### Data collection

The procedure that was employed by the researcher for getting the height and weight measurement of the children was as described by the WHO.^18^ was utilized for this study. The instruments were checked before each reading as a way of ensuring uniformity of reading, and the validity and reliability of the outcome.

### Data analysis

WHO AnthroPlus version 1.0.4^19^, a software for determining nutritional indices Z-scores which was developed by the WHO was used in obtaining the Height for age, Weight for age, and BMI for age Z-scores of the children. Children were classified as “underweight” (low weight for age, <−2 Z scores for children≤ 10 years old); “stunted” (low height for age, <−2 Z scores), “wasted” (low BMI for age < −2 Z scores), “overweight” (BMI for age Z score > +1 and ≤ +2), “obese” (BMI for age Z score > +2), and “normal weight” (BMI for age Z score −2 to + 1). Children with height-for-age Z-scores < −3 were defined as severely stunted. Those with BMI-for-age < −3 Z-scores were defined as severely wasted. Children with BMI-for-age Z-score > 3 were classified as obese.^20^

The data analysis was done using IBM Statistical Package for Social Sciences 20.0.^21^ Quantitative data like weight, height, and BMI were reported with their means and standard deviation (s.d.) while categorical data including; sex, school ownership and nutritional status were reported as proportions and percentages. Contrasts in the mean body weight, height, and BMI were assessed for male and female by age groups, utilizing an independent samples t test at 95% degree of confidence. Chi square test (*X*^2^) was utilized to test for between gender and nutritional status, age classifications and nutritional status, and school ownership and nutritional status. Although, the *X*^2^ test only indicates the absence or presence of associations between the categorical variables, frequency percentages between and across the variables helped to indicated the direction of these associations. The significant level was set at a probability level of p ≤ 0.05.

### Ethical considerations

This study was conducted with considerations to the rights and integrity of the research participants as provided by the Nigerian National Code of Health Research Ethics.22 Ethical clearance for this study was obtained from the Ondo State Health Research Ethics committee (OSHREC/01/07/19/137).

## Results

A total of 461 children (232 males, 229 females), who were aged 5–14 years old (mean age: 8.22 ± 1.82 years), were enrolled in the study. The mean height, weight, and BMI of the participants were 128.06 ± 9.91 cm, 26.14 ± 5.92 kg, and 15.73 ± 1.79 kg/m2, respectively. The modal class was age seven years with 54 males and 45 females. For the males in the modal class, the anthropometric values were as follows: Mean height was 122.35 ± 5.3 cm, mean weight was 22.72 ± 3.66 kg, and mean BMI was 15.1 ± 1.4 kg/m^2^. The corresponding measurements for females were 123.52 ± 5.72 cm, 23.00 ± 3.40 kg, and 15.02 ± 1.46 kg/m^2^.

In relation to age trends, children who were < 9 years of age had comparable height, weight, and BMI in the different gender categories. Notable differences in height, weight, and BMI were noted at the ages of 11, 12 and 13 years with females having higher values than their male counterparts, although most of these observed differences were not statistically significant.

The information presented in the first part of Table 2 is the proportion of school children at different cut-offs for Weight-for-Age Z scores (WAZ). Majority (83.6%) of the children had normal weight-for-age, 11.1% of them were overweight, 5% were underweight and one child (0.3%) was found to be severely underweight.

**Table 2:** Nutritional assessment of the school children

Indices	Frequency (*n*)	Percentage (%)
**Weight for Age**		
Overweight	38	11.1
Normal weight	286	83.6
Underweight	17	5.0
Severely underweight	1	0.3
**Total**	342	100
**Height for Age**		
Very tall	4	0.9
Normal height	335	72.7
Stunted	96	20.8
Severely stunted	26	5.6
**Total**	461	100
**BMI for Age**		
Obese	2	0.4
Severely overweight	4	0.9
Moderately overweight	29	6.3
Normal weight	315	68.3
Wasted	89	19.3
Severely wasted	22	4.8
**Total**	461	100

The second part of Table presents information on the Height-for-Age (HAZ) nutritional indicator. According to this sub-section, majority (72.7%) of the children fell within the normal height Z score, 20.8% were observed to be stunted, 5.6% were severely stunted, and four (0.9%) of the children were very tall.

The last part of the Table highlights information on the Body Mass Index-for-Age (BAZ) assessment. Here, majority (68.3%) of the children fell within the normal BAZ, 19.3% were wasted, 4.8% were severely wasted, and two (0.4%) children were found to be obese.

The association between the nutritional statuses of the subjects and their sex, school ownership and age category are presented in Table 3. Table 3 revealed that more males (85.8%) than females (81.5%) showed normal WAZ index, more females (13.9%) than males (8.3%) were found to be overweight. The finding was however, not statistically significant (p = 0.244). Most of the subjects were found to be in the normal height category of the HAZ index, more males (22.8%) compared to females (18.8%) were stunted, 5.6% of the total subjects were found to be severely stunted. The values for wasting and severe wasting were almost the same for both sexes. The majority of the subjects (68.3%) 68.5% males and 68.1% females showed normal weight on BAZ analysis.

**Table 3:** Association between nutritional status of subjects and gender

Variables	Gender		Total	*X* ^2^	*p*-value

	Male	Female			
**WAZ**					
Overweight	14 (8.3%)	25(13.9%)	39(11.1%)	4.171	0.244
Normal weight	145(85.8%)	141(81.5%)	286(83.6%)		
Underweight	10(5.9%)	7(4%)	17(5%)		
Severe underweight	0	1(0.6%)	1(0.3%)		
**Total**	169(100%)	173(100%)	342(100%)		
**HAZ**					
Very tall	1(0.4%)	3(1.3%)	4(0.9%)	2.879	0.411
Normal height	163(70%)	172(75.1%)	335(72.7%)		
Stunted	53(22.8%)	43(18.8%)	96(20.8%)		
Severely stunted	15(6.5%)	11(4.8%)	26(5.6%)		
**Total**	232(100%)	229(100%)	461(100%)		
**BAZ**					
Obese	1(0.4%)	1(0.4%)	2(0.4%)	4.145	0.529
Severely	0	4(1.7%)	4(0.9%)		
overweight					
Overweight	15(6.5%)	14(6.1%)	29(6.3%)		
Normal weight	159(68.5%)	156(68.1%)	315(68.3%)		
Wasted	46(19.8%)	43(18.8%)	89(19.3%)		
Severely wasted	11(4.7%)	11(4.8%)	22(4.8%)		
**Total**	232	229	461		

Table 4 showed that 83.8% of subjects in the age category 5-9 years compared to 77.8% recorded among subjects in the age category 10-15 years were found in normal weight on WAZ analysis. Slightly over eleven percent (11.1%) of the subjects in the age 10-15 years was found to be underweight compared to 4.1% underweight among children 5-9 years. Stunting was found among 33.3% and 16.4% of the subjects in the age 10-15 years and 5-9 years respectively. Significant association was found between the HAZ nutritional index and age category (p < 0.001). Wasting was found among 18.2% and 22.5% of the subjects in the age 5-9 years and 10-15 years, respectively.

**Table 4:** Association between nutritional status of subjects and age category

Variables	Age (years)		Total	*X^2^*	*p*-value

	5-9	10-14						
**WAZ**					
Overweight	37(11.1%)	1(11.1%)	38(11.1%)	0.766	0.858
Normal weight	279(83.8%)	7(77.8%)	286(83.6%)		
Underweight	16(4.8%)	1(11.1%)	17(5.0%)		
Severe underweight	1(0.3%)	0	1(0.3%)		
**Total**	333	9	342		
**HAZ**					
Very tall	4(1.2%)	0	4(0.9%)	24.782	0.000*
Normal height	267(78.3%)	68(56.7%)	335(72.7%)		
Stunted	56(16.4%)	40(33.3%)	96(20.8%)		
Severely stunted	14(4.1%)	12(10.0%)	26(5.6%)		
**Total**	341	120	461		
**BAZ**					
Obese	2(0.6%)	0	2(0.4%)	2.076	0.838
Severely overweight	3(0.9%)	1(0.8%)	4(0.9%)		
Overweight	23(6.7%)	6(5.0%)	29(6.3%)		
Normal weight	235(68.9%)	80(66.7%)	315(68.3%)		
Wasted	62(18.2%)	27(22.5%)	89(19.3%)		
Severely wasted	16(4.7%)	6(5.0%)	22(4.8%)		
**Total**	341	120	461		

* Significant value

From Table 5, 87.7% of all subjects from public schools had normal weight compared to 80.6% of the total children from the private schools. More subjects from private schools were overweight (16.3%), while more children from the public schools were underweight (8.2%). This WAZ index showed a statistically significant relationship with school type (p < 0.001). More of the subjects from private schools had normal height (82.4%) compared to those from the public schools (63.2%). Stunting was found among 30.3% of the subjects from public schools compared to 11.0% among the subjects from the private schools. This HAZ nutritional index was also determined to be significantly associated with School type (p<0.001) The BAZ analysis showed that more children from the public schools (23.9%) were wasted compared to those from the private schools (14.5%). The values for normal weight and severe wasting are comparable for both the public and the private schools. A statistically significant association was found between BAZ nutritional index and School type (P = 0.003).

**Table 5:** Association between nutritional status of subjects and school type

Variables	School type/ownership		Total	*X* ^2^	*p*-value

	Public school	Private school			
**WAZ**					
Overweight	6(4.1%)	32(16.3%)	38(11.1%)	17.891	0.000*
Normal weight	128(87.7%)	158(80.6%)	286(83.6%)		
Underweight	12(8.2%)	5(2.6%)	17(5.0%)		
Severe underweight	0	1(0.5%)	1(0.3%)		
**Total**	146	196	342		
**HAZ**					
Very tall	0	4(1.8%)	4(0.9%)	31.098	0.000*
Normal height	148(63.2%)	187(82.4%)	335(72.7%)		
Stunted	71(30.3%)	25(11.0%)	96(20.8%)		
Severely stunted	15(6.4%)	11(4.8%)	26(5.6%)		
**Total**	234	227	461		
**BAZ**					
Obese	0	2(0.9%)	2(0.4%)	17.698	0.003*
Severely overweight	0	4(1.8%)	4(0.9%)		
Overweight	8(3.4%)	21(9.3%)	29(6.3%)		
Normal weight	159(67.9%)	156(68.7%)	315(68.3%)		
Wasted	56(23.9%)	33(14.5%)	89(19.3%)		
Severely wasted	11(4.7%)	11(4.8%)	22(4.8%)		
**Total**	234	227	461		

* Significant value

## Discussion

This study conducted a baseline assessment of the nutritional status of school children in Ondo State Southwest Nigeria. This is done with the aim of providing a data on which the current NHGSFP and other subsequent nutrition intervention among the school children can be evaluated. Presentation the nutritional statuses of the school children from public (NHGSFP supported) schools and private (NHGSFP non-supported) schools were also made. Overall, we found that rates of stunting, wasting and obesity were low among the school children that were assessed (Table 2). The current nutritional status of the children is presented in the light of the SHP and the recent NHGSFP in Ondo State.

Although, the majority of the school children fell within the normal nutritional status the prevalence of the other forms of malnutrition among these children is also remarkable. The finding from this work where the bulk of the subjects presented normal nutritional statuses is line with the finding of Eze et al, in Enugu, Nigeria where it was reported that majority of the subjects were in the normal growth category.^13^

The report of Oninla et al, (2007) in a research that was carried out among school children in Osun State where it was reported that the overall prevalent rates of underweight, wasting and stunting were 61.2%, 16.8% and 27.6%, respectively^22^ is in contrast to our finding where the majority fall within the normal weight and height category (Table 2). The differences in findings may be connected to the fact that Oninla, et al study was conducted among public schools alone unlike this study that included subjects from private schools. Also, the Oninla et al (2007) study was conducted over 12 years (sufficient time for major phenomena e.g economic up-turn, increased standard of living of the people, increased minimum wages of workers etc., which can make the difference in the feeding pattern of families) prior to the current study.

The prevalence rates for wasting, underweight, and stunting for the current study were comparatively higher than the rates reported by Akor et al, (2010) in a study among school children in Jos Plateau where they reported 11.1% stunting, 10.3% underweight and 2.4% wasting.^23^ This lower rates of malnutrition as reported by Akor et al, is likely due to the fact that food substances are more available in the middle- belt region of Nigeria including Jos, Plateau state.^24^ Chinawa et al., (2021) conducted their own study on the assessment of the nutritional status of children with and without congenital heart disease, a prevalence rate of 23.4%, 9.5% and 8.2% were reported for wasting, stunting and overweight respectively. 24 This higher prevalence of wasting (23.4% compared to 19.3%). Even though the Chinawa et al., (2021) study is not comparable to the current study as it was conducted basically among hospital patients who were presented due to consequences of malnutrition, it reinforces the assertion that malnutrition is a major challenge among the Nigerian children, especially among the less privilege

A study from Ghana reported 19.4% wasting^25^, the same value for the current study while another report from Ethiopia documented a lower rate of 14.0% wasting^26^. These observed differences may be related to the study instruments such as the reference indices used, secular/ time trends, and sociocultural and economic factors.

There were notable gender variations in height, weight and BMI among the subjects age 11, 12 and 13 years with the female subjects having higher values than the males (Table 1), this finding corroborates the report of Eze et al, where it was reported that at age 10 years and above, female subjects were taller and heavier than their male counterpart^13^. Akor et al, reported the same finding^23^. This situation may be as a result of the fact that female children reach puberty faster than male children, the physiological changes in the body which comes with puberty resulting in additional weight gain makes the female children to be taller and bulkier as it has been confirmed by this study and other previous studies.

**Table 1:** Mean height, weight and body mass index of subjects

Age (years)	Sex	Freq	Mean Height (s.d.) in cm	*p*-value	Mean Weight (s.d.) in kg	*p*-value	Mean BMI (s.d.) in kg/m^2^	*p*-value
5	Male	12	115.25(7.20)	0.933	19.583(3.08)	0.362	14.664(1.13)	0.133
	Female	10	115.00(6.46)		18.500(2.17)		13.967(0.92)	
6	Male	36	118.75(4.81)	0.929	21.056(2.29)	0.665	14.913(1.20)	0.618
	Female	33	118.64(5.76)		21.424(4.49)		15.123(2.17)	
7	Male	54	122.35(5.32)	0.290	22.722(3.66)	0.698	15.101(1.44)	0.773
	Female	45	123.53(5.72)		23.000(3.39)		15.016(1.46)	
8	Male	32	126.50(6.03)	0.594	24.906(3.58)	0.188	15.510(1.41)	0.151
	Female	42	127.40(7.98)		26.310(5.10)		16.079(1.84)	
9	Male	35	132.66(6.01)	0.225	28.229(3.47)	0.922	16.006(1.40)	0.319
	Female	42	130.88(6.60)		28.333(5.48)		16.409(2.05)	
10	Male	31	135.84(6.20)	0.976	30.677(4.00)	0.843	16.594(1.59)	0.869
	Female	27	135.89(6.31)		30.926(5.50)		16.679(2.31)	
11	Male	22	137.59(7.33)	0.041	31.455(4.70)	0.226	16.534(1.61)	0.832
	Female	22	141.68(5.37)		33.000(3.57)		16.433(1.52)	
12	Male	6	137.00(9.45)	0.197	31.333(6.56)	0.485	16.497(1.49)	0.800
	Female	6	143.40(6.60)		33.333(1.63)		16.265(1.64)	
13	Male	2	146.00(1.41)	0.121	38.500(2.12)	0.333	18.074(1.35)	0.253
	Female	1	155.00(0.00)		34.000(0.00)		14.152(0.00)	
14	Male	2	151.00(2.12)	0.667	43.000(0.00)		18.740(0.53)	0.100
	Female	1	150.00(0.00)		33.000(0.00)		14.667(0.00)	
8.22 ±			128.06 ±		26.14 ± 5.92**†**		15.73 ± 1.79**†**	
1.82†			9.91**†**					

† Mean (± s.d.) of variable. Overall: xxx

Another study that compares between male and female nutritional status is that which was conducted by Alebiosu and colleagues where it was reported that the prevalence of underweight was higher in the male respondents than in their female counterparts^27^. This research finding corroborates the finding from this study even though Alebiosu and colleagues conducted their research among secondary school adolescents unlike this study that was conducted among primary school children that are younger in age. This finding underscores the need for adequate nutrition for the male children especially during puberty.

The BAZ assessment revealed that both male and female subjects presented equal proportion of normal weight (Table 3). The higher prevalence of stunting among the male subjects may be related to male children usually being more active thus requiring more nutrition, the shortage of which results in chronic conditions such as stunting. Younger children also show better nutritional status than the older children 10 years and above (Table 4). The two extremes of malnutrition were found more among the subjects 10-15 years (Table 4). This finding may be as a result of the fact that as these children advance in age, they require more nutrition to sustain their life activities including learning, the lack or shortage of which result in any of these malnutrition outcomes.

The HAZ assessment which is an indicator of chronic undernutrition shows that children from the private schools' fare better compared to their counterpart in the public schools whose prevalence of stunting almost tripled that which was found among the subjects from the private schools (Table 5). The other assessed nutritional index BAZ is comparable between the subjects from the public and the private schools. This finding is however surprising given the fact that the NHGSFP only covers children in the public schools with daily supply of meal during schooling hours. The seemingly better nutritional status of the subjects from the private schools may be as a result of better socioeconomic status of the parents of the children in the private schools which was not captured in this study. It is a common practice in the Southwest Nigeria for the rich to enrol their wards in the private schools, leaving the often time poorly funded public schools for the children of the poor.

## Conclusion

Majority of the children that were assessed were found to be doing good nutritionally and thus, healthy by extension. The proportion of malnourished children that were discovered is however worthy of note and at the same time worrisome. It was also surprising that current evidence revealed that those subjects from private schools who do not partake in the NHGSFP seem to present better nutritional status compared to subjects from the public schools where the NHGSFP has been ongoing for about five years, however, there was no baseline data to support this finding.

It is therefore, recommended that a further study be conducted where the findings from this study will be used as a baseline data on the nutritional statuses of the schoolage children in the study area. Also, steps should be taken by the government to make the NHGSFP more responsive to the actual nutritional needs of the school children so that its impact can be at least be felt by the children that are seemingly benefiting from the programme. In support of the efforts of the government at improving the nutritional indices among the school children, the parents should be trained by the health authority in the State on ways through which locally available food items can be appropriately mixed to supply children with the nutrients that they require for optimum growth. Review of the current school-feeding programme policy to cater for the children's actual nutritional requirements while considering the socio-economic status of the parents is recommended.
